# Afatinib combined with anti-PD1 enhances immunotherapy of hepatocellular carcinoma *via* ERBB2/STAT3/PD-L1 signaling

**DOI:** 10.3389/fonc.2023.1198118

**Published:** 2023-05-30

**Authors:** Chao Yu, Xinyi Zhang, Min Wang, Gaoxin Xu, Siqi Zhao, Yongheng Feng, Chao Pan, Weijun Yang, Jin Zhou, Longcheng Shang, Yong Ma

**Affiliations:** Department of General Surgery, Nanjing First Hospital, Nanjing Medical University, Nanjing, Jiangsu, China

**Keywords:** afatinib, hepatocellular carcinoma (HCC), PD-L1, immunotherapy, ERBB2

## Abstract

**Background:**

Afatinib is mainly used to treat advanced non-small cell lung cancer, but its therapeutic effect on hepatocellular carcinoma is still unclear.

**Methods:**

Over 800 drugs were screened by CCK8 technology and afatinib was found to have a significant inhibitory effect on liver cancer cells. The expression of PDL1 in tumor cells treated with drugs were detected by qRT-PCR and Weston Blot experiments. The effects of afatinib on the growth, migration and invasion of HCC cells were evaluated using wound healing, Transwell, and cell cloning assays. The in vivo effects of afatinib in combination with anti-PD1 were evaluated in C57/BL6J mice with subcutaneous tumorigenesis. Bioinformatics analysis was performed to explore the specific mechanism of afatinib's inhibition of ERBB2 in improving the expression level of PD-L1, which was subsequently verified through experiments.

**Results:**

Afatinib was found to have a significant inhibitory effect on liver cancer cells, as confirmed by in vitro experiments, which demonstrated that it could significantly suppress the growth, invasion and migration of HCC cells. qRT PCR and Weston Blot experiments also showed that Afatinib can enhance the expression of PD-L1 in tumor cells. In addition, in vitro experiments confirmed that afatinib can significantly enhance the immunotherapeutic effect of hepatocellular carcinoma. Afatinib’s ability to increase PD-L1 expression is mediated by STAT3 activation following its action on HCC cells.

**Conclusion:**

Afatinib enhances PD-L1 expression in tumor cells through the STAT3/PD-L1 pathway. The combination of afatinib and anti-PD1 treatment significantly increases the immunotherapeutic effect of HCC.

## 1 Introduction

Hepatocellular carcinoma (HCC) is the most common type of liver cancer and is the sixth most common cancer worldwide, with the second-highest mortality rate (1). The leading cause of HCC are hepatitis B virus (HBV) or hepatitis C virus (HCV) infection, chronic liver disease caused by alcoholism, and metabolic syndrome (2). Various treatments for HCC, such as liver transplantation, surgical resection, interventional therapy, targeted therapy, and immunotherapy, have been developed. However, surgery remains the only curative treatment option (3). Sorafenib, a multi-tyrosine kinase inhibitor (TKI), has been shown to prolong the survival period of advanced HCC (4). More recently, Lenvatinib, regorafenib and cabozantinib have also been found to significantly prolong the survival of HCC as first-line or second-line drugs (5–7).

In recent years, immune checkpoint inhibitors have emerged as a promising immunotherapy method for HCC treatment. Currently, the FDA has approved four immune checkpoint inhibitors for single or combined use as first-line or second-line treatment for HCC: atilizumab (targeting programmed death ligand 1 (PD-L1)) in combination with bevacizumab (targeting vascular endothelial growth factor (VEGF)), ipilimumab (targeting cytotoxic T lymphocyte-associated antigen 4 (CTLA-4)) in combination with nivolumab (targeting programmed cell death protein 1 (PD1)), and pembrolizumab (PD1) or nivolumab alone. However, the overall response rate of anti-PD1 treatment for HCC is only about 20%, suggesting limited benefits for most HCC patients (8). Hence, more comprehensive research is required to improve the therapeutic outcomes of anti-PD1 treatment for HCC.

The ERBB receptor is a family of receptor tyrosine kinase (RTK) consisting of four members: EGFR (also known as ERBB1), ERBB2 (HER2), ERBB3 (HER3) and ERBB4 (HER4) (9). These receptors are widely expressed in various tissues and play a critical role in regulating cell proliferation, differentiation, survival, and migration. Disorders in ERBB receptor-mediated signal transduction can cause diseases such as developmental disorders and cancer (10). Overexpression or over-activation of ERBB receptor, especially ERBB2, has been found in many types of cancer and has been used as an important drug target for the development of anticancer therapy. However, the role of ERBB2 in HCC immunotherapy is still unclear, and further research is needed.

In conclusion, our study has investigated the effect of ERBB2 on HCC cells and its potential clinical significance, as well as exploring the specific mechanism involved. These findings may offer new insights to guide future clinic practice.

## 2 Materials and methods

### 2.1 Drugs

All drugs were purchased from Selleck (USA).

### 2.2 Cell cultures

The human GC cell lines (HCCLM3 and Hep3B) were provided by the Shanghai Institutes for Biological Sciences in China. The cells were cultured in a DMEM medium (Gibco, Austria) supplemented with 10% fetal bovine serum (Gibco, Austria) and 1%penicillin-streptomycin, and maintained in a humidified incubator at 37°C with 5% CO2.

### 2.3 Cell counting kit-8 proliferation assay

The inhibitory effect of drugs on cell proliferation was evaluated using the CCK-8 kit (Ribobio, China). For drug screening, HCC cells (2000/well) were seeded in 96 well plates and incubated for 24 hours. The drugs to be tested were added at a concentration of 10 µ M per well. After 24 hours of treatment, cells were further incubated in a humidified incubator at 37° C with 5% CO2. Next, 10 µ l of CCK-8 reagent was added to each well and incubated for an additional 2 hours. The absorbance at 450nm was measured using an enzyme marker.

### 2.4 Quantitative reverse transcription polymerase reaction

Total RNA was extreated from cells using the centrifugal column method with the total RNA extraction kit (TIANGEN, China). The extracted RNA was reverse transcribed into cDNA using a reverse transcription kit (Vazyme, China), following the manufacturer’s instructions. Quantification of PD-L1 mRNA was performed using a SYBR PCR kit (Vazyme, China), and mRNA expression was normalized to GAPDH before calculation.

### 2.5 Western blot analysis

The anti PD-L1 antibody and anti β-actin antibody were purchased from Proteintech (USA), and total cellular protein was obtained by lysing cells in RIPA lysis buffer (Beyotime, China). A 5% skimmed milk powder solution was used for room temperature blocking for 2h. After incubating with anti GADPH and anti PD-L1 at 4° C for 12 hours, the membrane was incubated with secondary antibody (1:5000) and then detected using an enhanced chemiluminescence kit (NCM Biotech, China). The protein expression level was analyzed using ImageJ software to analyze the western blot image.

### 2.6 Wound healing assay

After the confluence of HCCLM3 or Hep3B cell lines reaches 90%, use the standard 200 µ l pipette tip to create a scratch quickly, and wash it twice with PBS to remove any floating debris. Then, replace with fresh culture medium containing10 µ M afatinib or dimethyl sulfoxide (DMSO) of the same concentration as a control. The plate is then placed in a humidified incubator at 37° C with 5% CO2. At different time points (0h, 24h, 48h), the wound healing within the scratch line is observed, and representative images of scratch line are taken.

### 2.7 Transwell assay

When the HCC cell line reached 90% confluency, 10 uM afatinib and an equal concentration of DMSO were added to the treatment and control groups, respectively, and then incubated in a humidified incubator containing 5% CO2 at 37° C for 24 hours. Following the manufacturer’s instructions, 4 × 106 HCC cells were added to the upper chamber with 200 µl DMEM medium containing 10uM afatinib, while the lower chamber contained 700 µl DMEM medium containing 10% fetal bovine serum as the chemotactic agent. The control group was treated with DMSO in the same way as the treatment group. After 24 hours, the upper chamber was fixed with formaldehyde and stained with 0.1% crystal violet. Finally, cell migration was observed and counted under the inverted microscope.

### 2.8 Colony formation assay

HCCLM3 and Hep3B cell lines were seeded into six well plates at a density of 1000 cells/well. The experimental group was treated with 10 uM afatinib, while the control group was treated with DMSO of the same concentration. The six-well plates were incubated in a humidified incubator at 37° C and 5% CO2 for 14 days. The cells were fixed with formaldehyde and stained with 0.1% crystal violet. Colonies were observed and counted under natural light, and representative wells were selected for images. All experiments were performed in triplicate.

### 2.9 Mice model

Animal experiments were conducted following the guidelines of the Animal Management Committee of Nanjing Medical University, and all animal procedures and management were in compliance with ethical provisions for animal experiments. Prior to the experiment, we randomly divided 20 4-week old male C57/BL6J mice into four groups: afatinib single group, anti-PD1 (BP0273, Biocell, USA) single group, afatinib+anti-PD1 combined group, and PBS group (n=5 for each group). Subcutaneous injection of H22 cells was performed in the mice. In the Afatinib group, we injected Afatinib intraperitoneally every 2 days (10mg/kg) for a total of 28 days. Anti-PD1 (5.5mg/kg) was injected intraperitoneally every 7 days for a total of 28 days. Intraperitoneal injection was performed at the same time and concentration as above in the dual-drug group. After the experiment, the mice were euthanized and the tumors were collected to measure their size and weight for further experiments.

### 2.10 Immunohistochemistry

Each tissue sample was stained with specific primary antibodies and biotin-conjugated secondary antibodies, followed by incubation with the antibody protein biotin peroxidase complex. The samples were evaluated using the H-score method, which combines the values of the intensity of immune response and the percentage of tumor cells stained. The final immunohistochemical score was obtained by multiplying the percentage of target protein positive cells by the intensity score.

### 2.11 ERBB2 expression level, clinicopathological analysis, promoter methylation level and pan-cancer analysis

UALCAN (http://ualcan.path.uab.edu/) was used to examine the differential expression of ERBB2 in HCC and normal tissues as well as the difference in promoter methylation level. Kaplan Meier plotter (kmplot.com) was used to determine the correlation between ERBB2 expression and recurrence free survival (RFS) as well as progression-free survival (PFS) in tumor patients. TIMER (cistrome.shinyapps.io/timer) was used to analyze the differential expression of ERBB2 in various tumor tissues.

### 2.12 Immune-related analysis of ERBB2

TISIDB (cis. hku. hk/TISIDB) is a portal that facilitates the study of the interactions between tumors and the immune system by integrating multiple heterogeneous data types. TISIDB was used to analyze the correlation between the abundance of tumor infiltrating lymphocytes (TIL) and ERBB2.

### 2.13 ERBB2 location analysis

The human protein atlas can be used to determine the location of ERBB2 in cells. The atlas provides a comprehensive map of the human proteome in various tissues and cell types, including information on protein expression, localization, and function. It also includes data on gene expression in normal and diseased tissues, as well as tools for visualizing and analyzing the data.

### 2.14 Gene pathway correlation analysis

RNA-sequencing expression (level 3) profiles and corresponding clinical information for LIHC were downloaded from the TCGA dataset (https://portal.gdc.com).The GSVA package in R software was used to perform gene set variation analysis, with the method parameter set to ‘ssgsea’. Spearman correlation was used to analyze the correlation between genes and pathway scores. All the analysis methods and R packages were implemented in R version 4.0.3. A p value <0.05 was considered statistically significant.

### 2.15 Statistical analysis

The continuous data obtained from a single test in the two groups were analyzed using Graphpad Prism 8.0.2 (United States). A p value<0.05 was considered statistically significant.

## 3 Results

### 3.1 Afatinib had significant inhibitory effect on liver cancer cells

**Figure 1:**
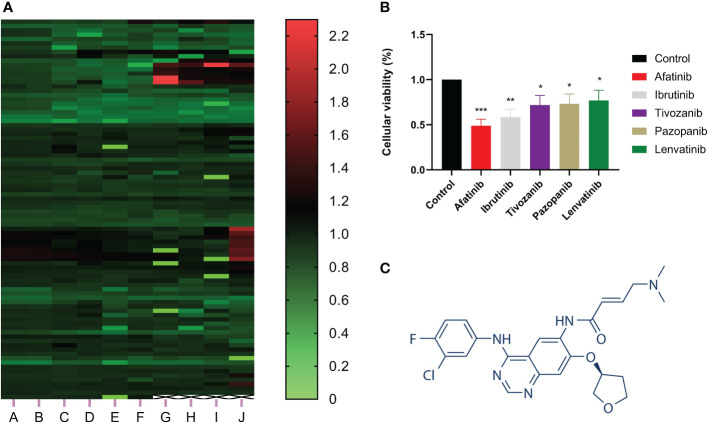
Afatinib significantly inhibited the proliferation of liver cancer cells among over 800 drugs. **(A)** Heat map showing the inhibitory effect of more than 800 drugs on the proliferation of liver cancer cells as determined by CCK8 experiment. **(B)** Comparison of the inhibitory effect of afatinib with four other commonly used chemotherapeutic drugs, Ibrutinib, Tivozanib, Pazopanib, and Lenvatinib, on the proliferation of liver cancer cells. **(C)** Chemical structure formula of Afatinib. * p < 0.05, ∗∗p < 0.01, ∗∗∗p < 0.001.

CCK-8 assay was utilized to investigate the effect of more than 800 drugs on the proliferation of hepatocellular carcinoma cells. The results showed that afatinib significantly inhibited the proliferation of HCC cells (**Figure 1A**). Additionally, we compared afatinib with other commonly used chemotherapeutic drugs including Ibrutinib, Tivozanib, Pazopanib, and Levatinib, and the results indicated that afatinib exhibited better inhibitory effect on HCC cells than other drugs (**Figure 1B**). The molecular structure formula of Afatinib is illustrated in **Figure 1C**. Therefore, we decided to further study the role and mechanism of afatinib in the treatment of HCC.

### 3.2 Afatinib can significantly inhibit the proliferation, invasion and migration of HCC cells

**Figure 2:**
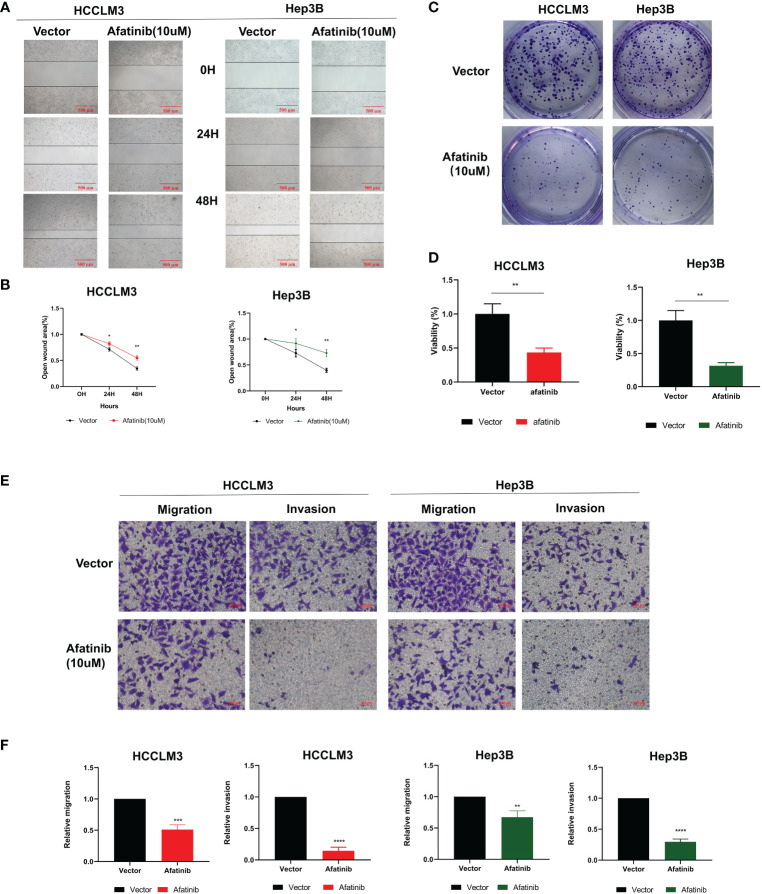
The ERBB2 inhibitor afatinib can inhibit the proliferation, invasion, and migration of HCC cells. **(A)** Wound healing assay was performed to assess the migration ability of HCC cells treated with 10 μM Afatinib. **(B)** Quantitation of the wound healing assay results. **(C)** Plate cloning assay was performed to investigate the proliferation ability of HCC cells treated with Afatinib at a concentration of 10 μM. **(D)** Quantitation of the plate cloning assay results. Afatinib can significantly inhibit the migration and invasion ability of HCC cells **(E)**: Transwell assay was used to assess the invasion and migration ability of HCC cells treated with Afatinib (10 μ M). **(F)** Quantitative graph of the results of the Transwell experiment. * p < 0.05, ∗∗p < 0.01, ∗∗∗p < 0.001, ∗∗∗∗p < 0.0001.

The inhibitory effect of afatinib on HCC cell migration was confirmed through wound healing assays in HCCLM3 and Hep3B cells at a concentration of 10 μM (**Figures 2A, B**). The colony formation assay also demonstrated that afatinib inhibited the proliferation of HCC cells (**Figures 2C, D**). Furthermore, the transwell assay revealed that afatinib significantly reduced the invasive and migratory potential of HCC cells compared to the control group (**Figures 2E, F**). These findings provide evidence for the potent inhibitory effect of afatinib on HCC cells.

### 3.3 ERBB2 is implicated in the clinical progress of HCC

**Figure 3:**
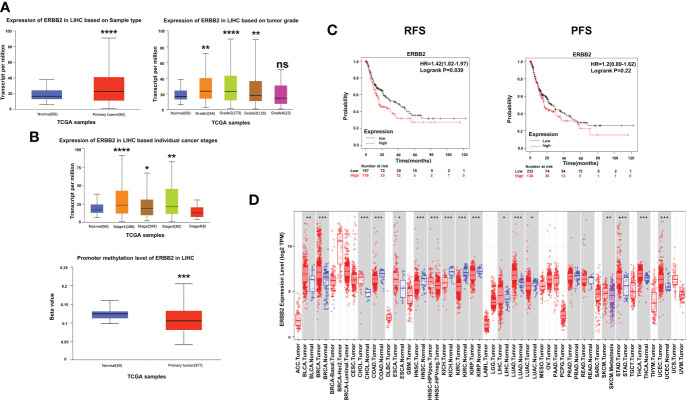
Expression of ERBB2 in clinical samples. **(A)** Comparison of ERBB2 expression levels between normal and tumor tissues of HCC; ERBB2 expression levels in different tumor grades; ERBB2 expression levels in different tumor stages. **(B)** Comparison of ERBB2 methylation levels between normal and tumor HCC tissues. **(C)** Kaplan Meier survival curve showed that patients with high ERBB2 expression have significantly worse RFS and PFS than those with low ERBB2 expression. **(D)** Pancancer analysis demonstrated differences in ERBB2 expression levels among different types of tumors. *p < 0.05, ∗∗p < 0.01, ∗∗∗p < 0.001, ∗∗∗∗p < 0.0001.

To further investigate the impact of ERBB2 inhibitor (afatinib) on the prognosis of HCC, we analyzed the Kaplan-Meier Plotter data and found that HCC patients with high ERBB2 expression had significantly lower recurrence free survival (RFS) and progression-free survival (PFS) than those with low or no expression (**Figure 3C**). TCGA portal analysis revealed that ERBB2 expression was significantly up-regulated in HCC tumor tissues compared to normal tissues, and the expression levels varied across different tumor grades and stages (**Figure 3A**). Furthermore, the methylation of the ERBB2 promoter was significantly lower in tumor tissues than in normal tissues (**Figure 3B**). Timer’s pancancer analysis also showed significant difference in ERBB2 expression between various common tumors and normal tissues (**Figure 3D**).

### 3.4 Expression of ERBB2 in human HCC tumor tissue

**Figure 4:**
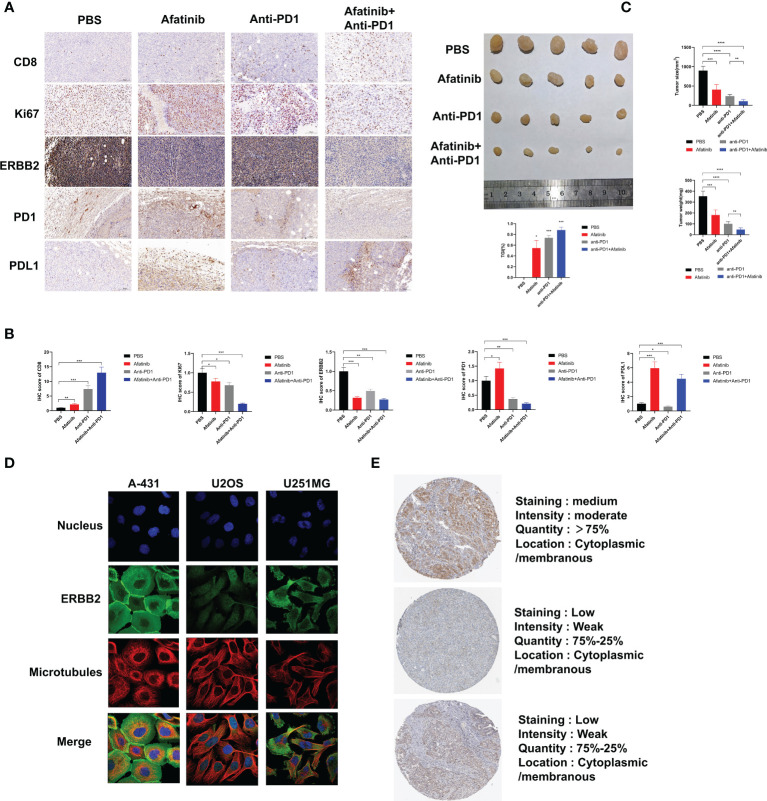
*In vivo* experiments have demonstrated that the ERBB2 inhibitor Afatinib can inhibit the development of HCC and enhance the therapeutic effect of anti-PD1 in HCC. **(A)** Immunohistochemistry was performed to assess the expression of CD8, Ki67, ERBB2, PD1, and PDL1 in PBS group, afatinib group alone, anti PD1 group alone, afatinib and anti-PD1 combined group. **(B)** The quantitative analysis of immunohistochemical results is shown. **(C)** The tumor weight and volume of subcutaneous tumors obtained from C57BL/6 mice were measured on the 28^th^ day. Expression of ERBB2 in various HCC cells and tissues. **(D)** Immunofluorescence staining shows the expression of ERBB2 in human epithelial squamous cell carcinoma A-431 cells, human osteosarcoma U2OS cells, and human glioma U251-MG cells. **(E)** Immunohistochemical staining of ERBB2 in HCC tissue samples. *p < 0.05, **p < 0.01, ***p < 0.001, ****p < 0.0001.

In addition, we analyzed the expression of ERBB2 in various malignant tumors using data from the Human Protein Atlas. Immunofluorescence analysis showed that ERBB2 was clearly expressed in the cytoplasm and cell membrane of human epidermal squamous cell carcinoma A-431 cells, human osteosarcoma U2OS cells, and human glioma U251-MG cells, indicating that ERBB2 expression was not limited to specific cell lines (**Figure 4D**). The Human Protein Atlas showed that ERBB2 staining in the tissues of HCC patients is mostly moderate or mild, and most HCC tissues express this protein, mainly distributed in the cytoplasm and cell membrane (**Figure 4E**). These findings suggest that ERBB2 is a promising target for the treatment of HCC.

### 3.5 Afatinib can significantly increase PDL1 expression in HCC tumor cells by up-regulating the STAT3

**Figure 5:**
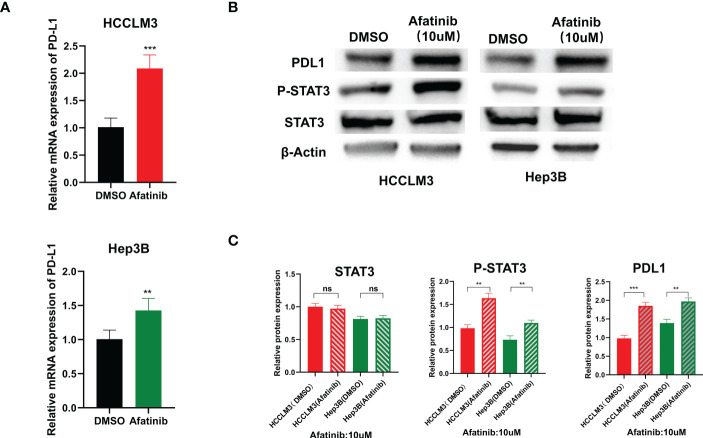
ERBB2 inhibitor afatinib upregulates the expression of PDL1 in HCC cells through STAT3. **(A)** qRT-PCR detected an increase in PDL1 expression in HCC cells treated with Afatinib. **(B)** Western blot analysis revealed an increase in PDL1 and p-stat3 expression in afatinib-treated cells. **(C)** Quantitative map of Western blot detection results. * p < 0.05, ∗∗p < 0.01, ∗∗∗p < 0.001. ns, not statistically.

We examined the expression level of PD-L1 in Hep3B and HCCLM3 hepatoma cell lines using qRT-PCR after treatment with afatinib for 24 hours. We found that the expression of PD-L1 increased in hepatoma cells after the addition of afatinib (**Figure 5A**). Consistently, the Western blot analysis also demonstrated an increase in PD-L1 protein expression in HCC cells treated with Afatinib for 24 hours (**Figure 5B**). To explore the underlying mechanism of the afatinib-induced increase in PD-L1, we referred to the research by Song et al. on T-cell lymphoma (11) and hypothesized that it could be related to STAT3. Therefore, we assessed the expression of STAT3 and P-STAT3 by Western blot, and as expected, the results showed no significant difference in STAT3 expression but a higher expression of P-STAT3 in the cells treated with afatinib compared to the control group (**Figures 5B, C**). In conclusion, our findings suggest that the ERBB2 inhibitor afatinib promotes the phosphorylation of STAT3, which in turn induces the increase in PD-L1 expression in HCC.

### 3.6 Afatinib combined with Anti-PD1 significantly increased the immunotherapeutic effect of HCC in *in vivo* model

We injected H22 cells into the bilateral subcutaneous groin of C57/BL6J mice (5 mice/group, 4 groups in total) at the age of 4 weeks, followed by intraperitoneal injection of PBS, afatinib, anti-PD1, or their combination to analyze the tumor growth size and weight for 28 days. The results showed that compared with the control group, the afatinib group inhibited the growth of HCC, while the anti-PD1 group significantly inhibited the development of HCC. As expected, the combined group of afatinib and anti-PD1 exhibited a better inhibitory effect on tumor growth compared to the single drug group and PBS control group. On day 28, the mice were euthanized, and tumor tissues were weighed and measured (**Figure 4C**). The tumor weight and volume of the afatinib and anti-pd1 single drug groups were lighter than those of the control group, while the combination of afatinib and anti-PD1 had a more significant tumor inhibition effect. Immunohistochemical analysis of HCC tumors was also performed (**Figures 4A, B**). The CD8 expression in both the single drug group and combination group was significantly higher than that in the control group, especially in the combination group of Afatinib and Anti-PD-1. In terms of Ki67 expression, both the single drug group and the combination group were able to reduce Ki67 expression, with the combination of Afatinib and Anti-PD1 showing the most significant reduction. Additionally, the results showed that the single drug group and the combination group had varying degrees of inhibitory effects on ERBB2 expression in HCC. The afatinib single drug group slightly increased the expression of PD-1, while the anti-PD1 and afatinib+anti-PD-1 combined group significantly reduced the expression of PD-1. Notably, the expression of PD-L1 was higher in the Afatinib monotherapy group and the combination of afatinib+Anti-PD1 group, highlighting the importance of targeting both ERBB2 and PD-1/PD-L1 pathways for HCC treatment. Overall, these findings suggest that the combination of ERBB2 inhibitor Afatinib and Anti-PD1 therapy is more promising approach for the treatment of HCC compared to traditional monotherapy.

### 3.7 The correlation between tumor infiltrating lymphocytes abundance and ERBB2 expression

**Figure 6:**
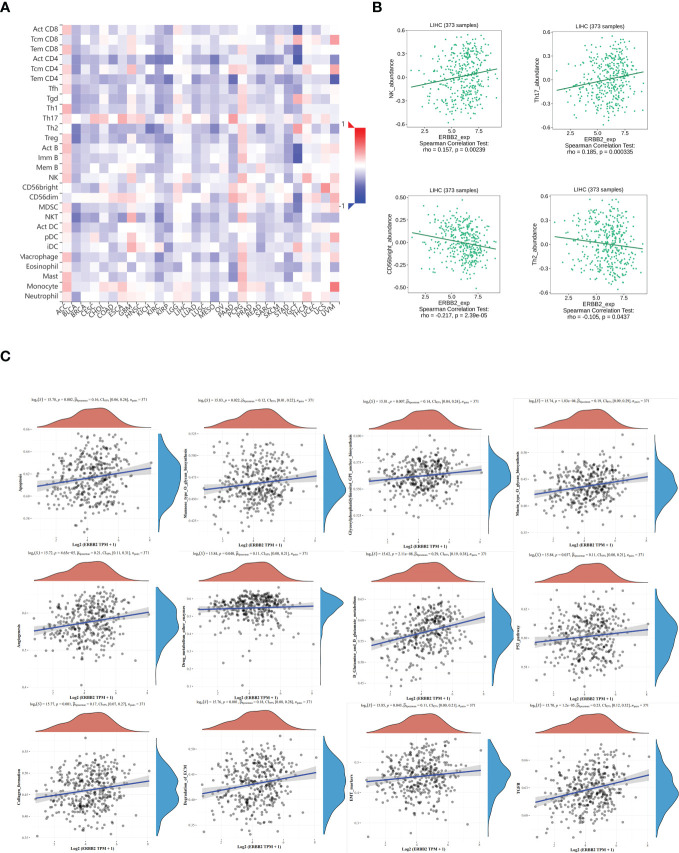
The relationship between tumor infiltrating lymphocytes (TIL) and ERBB2 expression in HCC. **(A)** Heatmap representing the infiltration abundance of multiple lymphocyte types in different tumors estimated by GSVA based on gene expression profiles. **(B)** Correlation analysis between ERBB2 expression and the abundance of NK, Th17, CD56bright, and Th2 in HCC. Bioinformatics analysis shows that ERBB2 participates in multiple cellular metabolic pathways of HCC, such as D-glutamine and D-glutamic acid metabolism, ECM degradation, EMT marker gene, p53 pathway, TGFb, mannose O-glycan biosynthesis, collagen formation, glycosylphosphatidylinositol GPI-anchored biosynthesis, cell apoptosis, angiogenesis, drug metabolism other enzymes, and mucin O-glycan biosynthesis **(C)**.

The relative abundance of tumor-infiltrating lymphocytes (TILs) was estimated using gene set variation analysis (GSVA) based on gene expression profiles of HCC (**Figure 6A**). Based on the immune-related characteristics of 28 TIL types in Charoentong’s study, we found that a large number of immune cell types were associated with ERBB2 expression. Specifically, NK/Th17 was significantly positively correlated with ERBB2, while CD56bright/Th2 was significantly negatively correlated with ERBB2 expression (**Figure 6B**). These results provide new clues and ideas for further immunotherapy of HCC.

### 3.8 ERBB2 participated in multiple metabolic pathways in HCC

Spearman analysis was used to evaluate the correlation between single genes and pathway score in HCC (**Figure 6C**). The X-axis represents the distribution of gene expression, while the Y-axis represents the distribution of pathway scores. The density curve on the right displays the trend of immune scores of the pathway, and the curve on the top shows the trend of gene expression. The value on top indicates the p value, correlation coefficient, and correlation calculation method. We found that ERBB2 was significantly correlated with several pathways in HCC, including D-glutamine and D-glutamic acid metabolism, ECM degradation, EMT marker gene, p53 pathway, TGFb, mannose O-glycan biosynthesis, collagen formation, glycosylphosphatidylinositol GPI-anchored biosynthesis, cell apoptosis, angiogenesis, drug metabolism other enzymes, and mucin O-glycan biosynthesis.

## 4 Discussion

HCC is one of the most common malignant tumors worldwide, with approximately 1 million new cases occurring each year. Although surgical resection and liver transplantation are considered effective methods for treating HCC, but the recurrence rate of HCC remains high. In recent years, with the development of numerous targeted and immunotherapies, there are now more options available for the treatment of HCC.

After screening over 800 small molecule drugs, we identified afatinib, an orally effective and irreversible ERBB2 inhibitor, as having a significant inhibitory effect on HCC cells. Although Afatinib is mostly used in the research of esophageal squamous cell carcinoma (ESCC), non small cell lung cancer (NSCLC), and gastric cancer, its efficacy in treating HCC was not clear. Previous studies suggested that ERBB2 was rarely expressed in HCC, leading to speculation that it may not play a role in HCC (12, 13). Deepak Parashar et al. found that in ovarian cancer, ERBB2 up-regulates ZEB1 in non-attached cells, FOX1 up-regulates ZEB1, and ZEB1 inhibits FOXM1 expression through a negative feedback mechanism. The authors suggest that ZEB1 and FOXM1 are significant in ERBB2 signaling for peritoneal metastasis of ovarian cancer (14). Anjali Geethadevi1 et al. found that in ovarian cancer by RNA seq, Binding of OSM to OSMR caused OSMR-IL6ST dimerization, which is required to produce oncogenic signaling cues for prolonged STAT3 activation (15). Yingqing Deng et al. found that, Single-cell sequencing in high-grade serous ovarian cancer (HGSOC) has shown that ERBB2 is highly expressed in metastatic tumors, and IL6/STAT3 signaling is enriched in fibroblasts and involved in angiogenesis and immune regulation (16). Deepak Parashar et al. found that genomic amplification of 3q26.2 locus leads to the increased expression of microRNA 551b-3p (miR551b-3p) in triple-negative breast cancer (TNBC). MiR551b upregulates the expression of Oncostatin M receptor (OSMR) and interleukin-31 receptor-α (IL31RA) as well as their ligands Oncostatin-M (OSM) and interleukin 31 (IL31) through STAT3 transcription (17). Ling Gao et al. found that in astrocytoma, the inhibition of STAT3 and ERBB2 significantly reduced the survival of glioma cells after radiation and inhibited tumor growth *in vivo*, which they believed was related to the mitochondrial apoptotic pathway (18). However, Shi et al. (19–21) conducted detailed studies that confirmed the expression and function of ERBB2 in HCC, prompting us to explore its specific mechanism. Through wound healing, cloning, and Transwell experiments, we confirmed that the ERBB2 inhibitor afatinib significantly inhibited the migration, proliferation, and invasion of HCC cells. We conducted qRT-PCR and WB experiments, which revealed that treatment of HCC cells with afatinib significantly increased the expression of PDL1. By reviewing previous studies, we found that STAT3 played a role in the expression of PDL1 in HCC. We also confirmed through WB experiments that after afatinib treatment, the expression of phosphorylated STAT3 was upregulated, resulting in an upregulation of PDL1 in HCC. However, the specific mechanism underlying this effect remains to be explored in future studies. To investigate the clinical relevance of ERBB2, we used UALCAN to analyze the expression of ERBB2 in HCC tumor tissues compared to normal tissues. Our results showed that ERBB2 expression was significantly higher in HCC tumor tissues than normal tissues. Furthermore, the expression levels of ERBB2 were higher in grade 1 and grade 2 tumors as well as in stage 1, 2, and 3 tumors when compared to normal tissues. However, the methylation of REBB2 was found to be lower in tumor tissues than in normal tissues. Additionally, Kaplan Meier Plotter analysis confirmed that patients with high ERBB2 expression had poorer survival and prognosis. Pancancer analysis revealed differential expression of ERBB2 across various tumor tissues. Furthermore, through data mining of The Human Protein Atlas, we observed a relatively fixed distribution of ERBB2 in cells. The immunohistochemical analysis of tumor tissue revealed variable expression of ERBB2 in HCC, and the location of the expressed cells was consistent with the results of immunofluorescence analysis. In order to further validate our conclusion that afatinib enhances HCC immunotherapy by upregulating PDL1, we conducted *in vivo* experiments in mice. Our results demonstrated that the combination of afatinib and anti-PD1 significantly reduced tumor volume and weight compared to the control group and the single drug group. We also confirmed the reliability of our experiments and conclusions through immunohistochemical experiments.

Through bioinformatics analysis, we investigated the association between TIL abundance and ERBB2 expression in HCC and observed a positive correlation between NK/Th17 cells and ERBB2, while a negative correlation was found between CD56bright/Th2 cells and ERBB2 expression. In addition, we analyzed the involvement of ERBB2 in various cellular functional pathways in HCC, which could potentially offer novel avenues for further research in this field.

**Figure 7:**
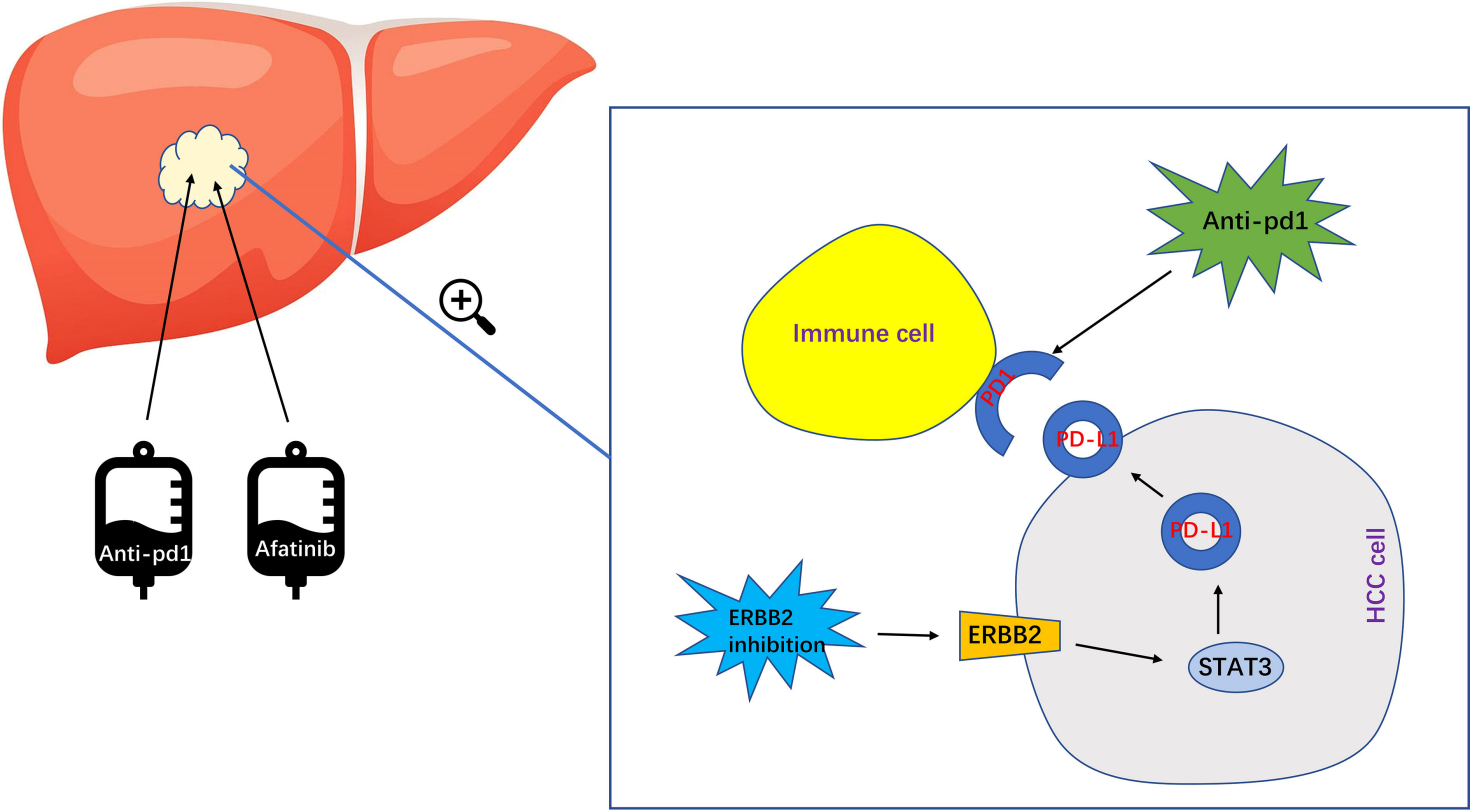
The specific mechanism of ERBB2 inhibitor Afatinib combined with anti PD1 to enhance the immunotherapeutic effect of HCC. Afatinib inhibits ERBB2, which leads to the upregulation of activated STAT3 expression. This in turn upregulates the expression of PDL1, ultimately enhancing the immunotherapy of HCC.

In summary, while research on ERBB2 in HCC is still relatively limited, our study has found that the combination of ERBB2 inhibitor Afatinib with Anti pd1 can significantly enhance the therapeutic effect of HCC (**Figure 7**). However, further studies are needed to elucidate the specific molecular mechanisms involved. With the development of immunotherapy for HCC in recent years, there is great potential for further exploration and advancement in this field.

## Data availability statement

The datasets presented in this study can be found in online repositories. The names of the repository/repositories and accession number(s) can be found in the article/**Supplementary Material**.

## Ethics statement

The animal study was reviewed and approved by Ethics Committee of Nanjing Hospital Affiliated to Nanjing Medical University.

## Author contributions

There are four first authors in this manuscript, who have made the equal contribution to this project. CY and XZ is responsible for writing the manuscript and experiment. MW and GX are responsible for the experiment. SZ, YF, CP and WY also participated in data collation and chart making. In addition, there are three corresponding authors. YM contributed to the key revisions of the research design and manuscript. JZ and LS are responsible for proofreading manuscripts and data. All authors contributed to the article and approved the submitted version.
